# Amyotrophic lateral sclerosis stratification: unveiling patterns with virome, inflammation, and metabolism molecules

**DOI:** 10.1007/s00415-024-12348-7

**Published:** 2024-04-21

**Authors:** Elena Niccolai, Matteo Pedone, Ilaria Martinelli, Giulia Nannini, Simone Baldi, Cecilia Simonini, Leandro Di Gloria, Elisabetta Zucchi, Matteo Ramazzotti, Pietro Giorgio Spezia, Fabrizio Maggi, Gianluca Quaranta, Luca Masucci, Gianluca Bartolucci, Francesco Claudio Stingo, Jessica Mandrioli, Amedeo Amedei

**Affiliations:** 1 Department of Experimental and Clinical Medicine, University of Florence, Florence, Italy; 2 Department of Statistics, Computer Science, Applications “G. Parenti”, University of Florence, Florence, Italy; 3 Neurology Unit, Department of Neuroscience, Azienda Ospedaliero Universitaria Di Modena, Modena, Italy; 4 Department of Experimental and Clinical Biomedical Sciences “Mario Serio”, University of Florence, Florence, Italy; 5 Department of Translational Research, Retrovirus Center - University of Pisa, Pisa, Italy; 6 Laboratory of Virology, National Institute for Infectious Diseases Lazzaro Spallanzani – IRCCS, Rome, Italy; 7 Department of Laboratory and Infectious Sciences, A. Gemelli University Hospital IRCCS, Rome, Italy; 8 Department of Neurosciences, Psychology, Drug Research and Child Health Section of Pharmaceutical and Nutraceutical Sciences, University of Florence, Florence, Italy; 9 Department of Biomedical, Metabolic and Neural Sciences, University of Modena and Reggio Emilia, Modena, Italy

**Keywords:** Amyotrophic lateral sclerosis, Cytokines, Short chain fatty acids, TTV, Virome, Metabolism, Microbiome

## Abstract

Amyotrophic lateral sclerosis (ALS) is an untreatable and clinically heterogeneous condition primarily affecting motor neurons. The ongoing quest for reliable biomarkers that mirror the disease status and progression has led to investigations that extend beyond motor neurons’ pathology, encompassing broader systemic factors such as metabolism, immunity, and the microbiome. Our study contributes to this effort by examining the potential role of microbiome-related components, including viral elements, such as torque tenovirus (TTV), and various inflammatory factors, in ALS. In our analysis of serum samples from 100 ALS patients and 34 healthy controls (HC), we evaluated 14 cytokines, TTV DNA load, and 18 free fatty acids (FFA). We found that the evaluated variables are effective in differentiating ALS patients from healthy controls. In addition, our research identifies four unique patient clusters, each characterized by distinct biological profiles. Intriguingly, no correlations were found with site of onset, sex, progression rate, phenotype, or C9ORF72 expansion. A remarkable aspect of our findings is the discovery of a gender-specific relationship between levels of 2-ethylhexanoic acid and patient survival. In addition to contributing to the growing body of evidence suggesting altered peripheral immune responses in ALS, our exploratory research underscores metabolic diversity challenging conventional clinical classifications. If our exploratory findings are validated by further research, they could significantly impact disease understanding and patient care customization. Identifying groups based on biological profiles might aid in clustering patients with varying responses to treatments.

**Graphical abstract:**

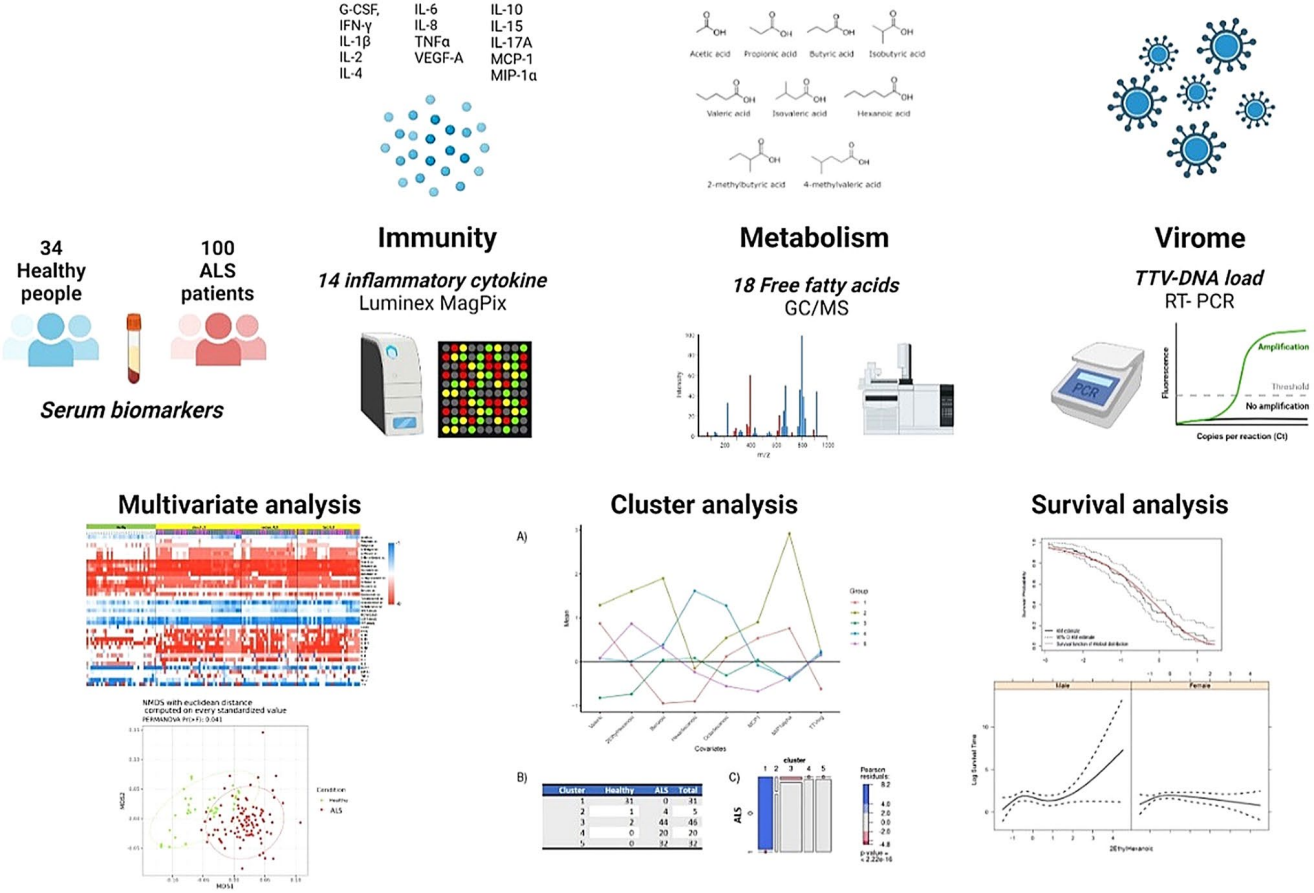

**Supplementary Information:**

The online version contains supplementary material available at 10.1007/s00415-024-12348-7.

## Introduction

Amyotrophic lateral sclerosis (ALS) is a neurodegenerative disease affecting motor neuron (MN), marked by significant genetic and clinical heterogeneity. This diversity mirrors the disease’s biological complexity and the not entirely understood pathomechanisms behind its progression. ALS heterogeneity has implications for patient counseling, individual prognosis assessment, participant stratification in clinical trials, the development of new therapeutic strategies, and the timing of treatment interventions and care management [28]. A significant aspect of ALS’s complexity is the recognized contribution of non-cell autonomous toxicity, with growing evidence highlighting the role of systemic factors such as metabolism and immunity [1]. These factors are not only involved in the multistep process of ALS development, but also influence the pace of disease progression [48]. Their accessibility presents them as potential targets for research into diagnostic and prognostic biomarkers [47]. The underlying causes behind metabolic dysfunction, immune regulation failure, and sustained neuroinflammation in ALS remain mostly unknown.

The microbiome metabolites and components are essential for maintaining immune homeostasis [56]. It is acknowledged that the microbiome substantially affects brain physiological processes, altering host susceptibility to various disorders, including neurodegenerative diseases [68]. Specifically, the gut microbiota impacts the central nervous system (CNS), through a bidirectional interaction, the gut–brain axis, influencing neuronal health via the production of neuroactive metabolites such as short chain fatty acids (SCFA) and toxins, and modulates the immune system [39], for instance, by affecting T cells’ activity and differentiation [13, 54]. In addition, the viral components of the microbiome, known as the “virome,” may have a significant role in maintaining immune health [8]. The human virome comprises a variety of commensals and pathogenic viruses that elicit a broad range of immune responses from the host. While persistent viral immunomodulation is associated with several inflammatory disorders, beneficial effects, such as protection against diseases, have also been observed [51]. The most common element of the human virome is the torque teno virus (TTV), belonging to the Anellovirus family, which causes persistent human infections [32, 66]. Levels of TTV viremia, especially high in immunosuppressed patients [19, 21, 26, 57], have been closely associated with host immunity, suggesting their potential as biomarkers for assessing the immune system functionality [40, 55]. Given the significant inter-individual variations in immunity and disease susceptibility influenced by the composition and function of the human microbiome, our study aims to enhance the understanding of ALS through microbiome, metabolism, and immune-derived molecules to characterize patients. Our findings unveil distinct patient clusters based on biological variables and identify a gender-specific association with patient survival, providing new insights into ALS pathogenesis and potential therapeutic approaches.

## Materials and methods

### Study population

We conducted a case–control cohort study at the ALS Center of Modena University Hospital in Italy, involving 100 patients with newly diagnosed ALS between January 2017 and January 2020, with follow-up extending to July 2022. The ALS Center of Modena coordinates the Register of ALS of Emilia Romagna region (ERRALS) [23, 44], which encompasses a population of 4.5 million inhabitants. The patients were diagnosed with possible, probable, or definite ALS, according to the Revised El Escorial criteria [7]. Thirty-four healthy individuals were also recruited among healthy unrelated spouses of patients. Both the patients and the healthy controls had to be aged between 18 and 80 years, have a BMI of 18 or above, and possess the capacity to understand and provide informed consent. The exclusion criteria included dementia or any other condition that compromised the ability to consent; known organic gastrointestinal disease (including, but not limited to, malignancy, inflammatory bowel disease, gastric ulcer, chronic diarrhea, gastroesophageal reflux); celiac disease and/or documented food intolerances (e.g., lactose intolerance); autoimmune disorders; severe comorbidities (such as liver, heart, or kidney failure, or chronic infections such as HIV, TBC, or hepatitis); history of complicated gastrointestinal surgery; and acute infections at the time of sampling. The study received approval from the Ethical Committee of Modena (Comitato Etico Provinciale di Modena, file n. 15/17), and informed consent was obtained from all participants. The reporting of clinical data complies with the STROBE guidelines.

### Clinical data

Clinical data were obtained from ERRALS, which has been enrolling MND patients at the time of their diagnosis and prospectively collecting demographic and clinical information since 2009 [44]. For all participants in this study, we extracted details on sex, age, and site of symptom onset (bulbar, upper limb or lower limb, respiratory), date of diagnosis, phenotype (classic, bulbar, upper motor neuron predominant ALS, flail arm, and flail leg, respiratory) [9, 58], genotype, and the ALS Functional Rating Scale-Revised (ALSFRS-R) total score at the time of diagnosis, at sampling and at the last available observation [43]. Except for six patients, all were screened at least for mutation in *C9ORF72, SOD1, FUS,* and *TARDBP* as detailed elsewhere [45]. Participants were followed from diagnosis until death or the last observation, whichever occurred first. The dates of initiation for nutritional or respiratory support were also recorded. Survival was measured from disease onset to death or the commencement of invasive ventilation. The rate of disease progression at diagnosis was determined by subtracting the ALSFRS-R score from 48 (the value used for symptom onset) and dividing by the number of months until diagnosis [33]. Disease progression rate was also calculated at the time of sampling and at the last observation, considering the monthly decline in the ALSFRS-R score from the symptom onset and diagnosis. Disease progression was classified as “slow” if ≤ 0.4 points per month, “fast” if ≥ 1 point per month, and “intermediate” for rates in between.

### Sample collection and processing

Serum samples were obtained by venipuncture at the time of diagnosis or shortly thereafter, following an overnight fasting period, and processed following standard procedures. After sample centrifugation for 10 min at 1300 × g, the supernatant was divided into aliquots and stored in polypropylene tubes at –80 °C in Modena Neurobiobank, until shipping to University of Florence.

### Evaluation of inflammatory molecules

We evaluated the serum levels of 14 cytokines by Milliplex MAP kits (Human Cytokine/Chemokine/Growth Factor Panel A, Magnetic Bead Panel) for Luminex MAGPIX detection system (Merck KGaA, Darmstadt, Germany) and following the manufacturers’ instructions. More specifically, we analyzed granulocyte colony-stimulating factor (G-CSF), interferon (IFN)-γ, interleukin (IL)-1β, IL-2, IL-4, IL-6, IL-8, IL-10, IL-15, IL-17A, monocyte chemotactic protein 1 (MCP-1), macrophage inflammatory protein-1α (MIP-1α), tumor necrosis factor-α (TNFα), and vascular endothelial growth factor (VEGF)-A. The levels of cytokines were estimated using a 5‐parameter polynomial curve (Bio-Plex Manager software), and the results were managed with Bio-Plex DataPro Software (Bio-rad Laboratories Inc.).

### Serum free fatty acid (FFA) quantification

The serum levels of short chain (SCFA: acetic, propionic, butyric, iso-butyric, iso-valeric, valeric, 2-methylbutyric, and hexanoic acids), medium chain (MCFA: heptanoic, nonanoic, 2-ethylhexanoic, octanoic, decanoic, benzoic, and dodecanoic acids), and long chain (LCFA: tetradecanoic, hexadecanoic, and octadecanoic acids) fatty acids were determined by gas chromatography coupled with mass spectrometry. The standard curves’ preparation was performed by an Agilent GC–MS 114 system composed of a 5971 single-quadrupole mass spectrometer, 5890 gas chromato-115 graph, and 7673 autosampler, as previously described [3].

### Quantification of TTV-DNA plasma levels

Viral DNA was extracted from 200 μl of serum using QIAamp DNA Mini kit (QIAGEN, Chatsworth, CA) and used to determine the presence and loads of TTV-DNA using a single-step universal TaqMan real-time PCR assay [41]. This assay uses primers (AMTS, 5′-GTGCCGIAGGTGAGTTTA-3′; AMTAS, 5′-AGCCCGGCCAGTCC-3′) and probes (AMTPTU, 5′-TCAAGGGGCAATTCGGGCT-3′) designed on a highly conserved segment of the untranslated region of the viral genome and has, therefore, the capacity to detect all the species in which TTV is classified. TTV loads were expressed as the number of viral DNA copies/mL of serum sample. The lower limit of detection was 10 copies of TTV-DNA/mL. The procedures used to quantitate the copy numbers and assess specificity, sensitivity, intra- and inter-assay precision, and reproducibility have been previously described [41]. All the procedures to validate the amplification process and to exclude the presence of carryover contaminations were performed: serum handling, DNA extraction, PCR amplification, and electrophoresis analysis were carried out in independent rooms; appropriate negative controls were added during DNA extraction and PCR amplification; and positive and negative controls (i.e., no template control and/or no amplification control) were run in each PCR.

### Statistical analysis

Categorical variables were presented as absolute frequencies and percentages and were compared between ALS patients and healthy controls using the *χ*^2^ test for unpaired data. Continuous variables were presented as median value and interquartile range (calculated as difference between the 75th and 25th percentiles of the data) and were compared by means of Mann–Whitney *U*-test (Bonferroni correction) in SPSS 27.0. *P* values less than 0.05 were considered statistically significant.

High-throughput data are often subject to batch effects. We employed the ComBat method proposed by Johnson et al. [31] to remove known batch effects due to experiments conducted under different conditions. ComBat is robust to outliers even in small batch sizes. Batch effects adjustment was performed with R package “sva” [37].

We employed the permutational multivariate analysis of variance (PERMANOVA) to compare multivariate sample means across different groups. PERMANOVA is a statistical test that does not rely on distributional assumptions of the data (i.e., normality), making it better suited than traditional methods for analyzing complex data. In addition, we used non-metric multidimensional scaling (NMDS) to simplify multivariate data into a few relevant axes to facilitate recognition and interpretation of nonlinear patterns and differences among groups. These plots have been computed on Euclidean distance using R 4.2 with the help of the packages vegan 2.6.2 and ggplot2 3.3.6 to compare groups.

### Cluster and survival analysis

A Gaussian Mixture Model (GMM) [4] was implemented to identify common biological profiles among the subjects. GMM is a model-based approach to clustering that associates each component of a finite mixture with a cluster. To focus our analysis on features that presented a large informative power, we retained in the analysis only those features whose median absolute deviation (MAD) exceeded a significance threshold. In fact, a larger MAD corresponds to a higher discriminatory power. All the continuous variables have been standardized. This method produces clusters of subjects that are homogeneous in terms of biological features, and each cluster is modeled with a multivariate Gaussian distribution. We select the number of clusters by Bayesian Information Criterion (BIC). The method’s output is a list of clusters. The method allocates to each cluster similar subjects (in terms of biological features) and also estimates the parameters of the multivariate Gaussian density associated with the cluster. GMM analysis is performed with R package “Mclust” [59]. For the survival analysis, we adopted a two-stage analysis to estimate the associations between survival time and biological features. In the first step, we deployed a modified version of the sure independence screening (SIS) [16] procedure. SIS uses the notion of marginal correlation––in our case, the correlation of a single biological feature with the survival time––to rank the features. We selected for step 2 those features with the smallest *p*-value from an accelerated failure time (AFT) [67] model with that given feature as the only predictor. An AFT model is a parametric model that measures the impact of the predictors on the survival time (instead of hazard, as in the Cox model). In the AFT model, the effect of explanatory variables is to accelerate or decelerate the time to event by a constant factor. In this study, for each predictor, we estimated the p-value testing the model with the considered feature plus the intercept against a model with only the intercept with a likelihood-ratio test. In the second step, we used the AFT model to determine the joint effect of the selected biological features and clinical factors on survivalIn particular, and the underlying assumption is that the ${\text{log}}$ of the survival time is linearly affected by the biological features. All the statistical procedures for cluster and survival analysis were performed in R.

## Results

### Clinical and demographical characteristics of participants

We analyzed a cohort of 100 patients with newly diagnosed ALS and 34 sex-matched healthy controls as depicted in the study diagram (Supplementary Fig. 1). Patients’ features are detailed in Supplementary Table 1. The average age at the time of sampling was 67 years (ranging from 37 to 93) for patients and 70 years (range 51–81) for HC (*P* = 0.146). Among the patients, 28 presented bulbar symptoms, 69 had limb onset, and 3 exhibited early respiratory impairment. Forty-six patients developed a classic phenotype, 25 bulbar, 18 flail arm/leg, 7 pyramidal, and 3 respiratory phenotypes. Disease progression, as indicated by the initial monthly decline in ALSFRS-R scores, was slow in 42 patients, fast in 31, and intermediate in 27. Genetic analysis revealed C9orf72 expansion in six patients and a FUS mutation in one. No other mutations were detected among the remaining patients, excluding six cases that were not genetically analyzed. The average ALSFRS-R total score at the time of sampling was 41.18 (SD:5.78). During the follow-up, 73 out of the 100 ALS patients either died or underwent tracheostomy with a mean survival time of 38.56 (SD:31.15) months from symptom onset.

### Cytokine levels

Analysis revealed a distinct cytokine profile in ALS patients, with 10 out of 14 tested cytokines showing lower expression in ALS patients compared to healthy controls and IL-8 (CXCL8) being more highly expressed in ALS patients relative to healthy controls (Table 1).

**Table 1:** Concentrations of cytokines in the serum of ALS patients and healthy controls

Analyte (pg/ml)	ALS patients (*n* = 100), median (IQR)	Healthy controls (*n* = 34), median (IQR)	*P* value (Mann–Whitney test)
G-CSF	7.77 (2.03–14.03)	18.45 (11.33–18.45)	< 0.0001*
IFN-γ	1.36 (0.62–2.66)	3.69 (1.0975–3.69)	0.0120
IL-10	1.47 (0.61–3.45)	3.88 (3.29–3.88)	< 0.0001*
IL-15	0.88 (0.54–2.02)	0.32 (0.26–0.32)	< 0.0001*
IL-17A	1.69 (0.53–4.74)	0.695 (0.32–0.695)	0.011
IL-1β	0.36 (0.36–1.56)	5.27 (1.48–5.27)	< 0.0001*
IL-2	0.48 (0.48–1.57)	9.24 (6.915–9.24)	< 0.0001*
IL-4	2.51 (2.51–3.30)	5.57 (1.63–5.57)	0.7741
IL-6	0.40 (0.40–3.44)	5.53 (3.6425–5.53)	< 0.0001*
IL-8	10.85 (5.87–17.79)	1.38 (1.38–1.38)	< 0.0001*
MCP-1	340.80 (208.26–444.78)	679.73 (571.525–679.73)	< 0.0001*
MIP-1α	3.57 (1.61–5.90)	15.41 (9.67–15.41)	< 0.0001*
TNF-α	9.54 (3.29–14.68)	17.04 (8.685–17.04)	0.003*
VEGF-A	43.76 (4.12–97.95)	469.469 (206.245–469.469)	< 0.0001*

*P*-values were calculated with Mann–Whitney test; ^*^p-value adj < 0.0035

No significant differences in cytokine expression were observed across patients with different disease onset, progression rates, phenotypes, or genotypes (including the presence of *C9ORF72* expansion or *FUS* mutation) (Supplementary Table 2).

### TTV-DNA status

ALS patients displayed a significantly higher serum load of TTV-DNA compared to healthy controls: TTV-DNA was detected in 88 out of 100 (88%) ALS serum samples with a mean TTV load of 2,370 (range 180–15,930) copies/mL, while in the control group, 24 out of 34 (71%) serum samples tested positive for TTV, with a mean load of 157 (11–750) copies/mL (*P* < 0.0001).

**Fig. 1:**
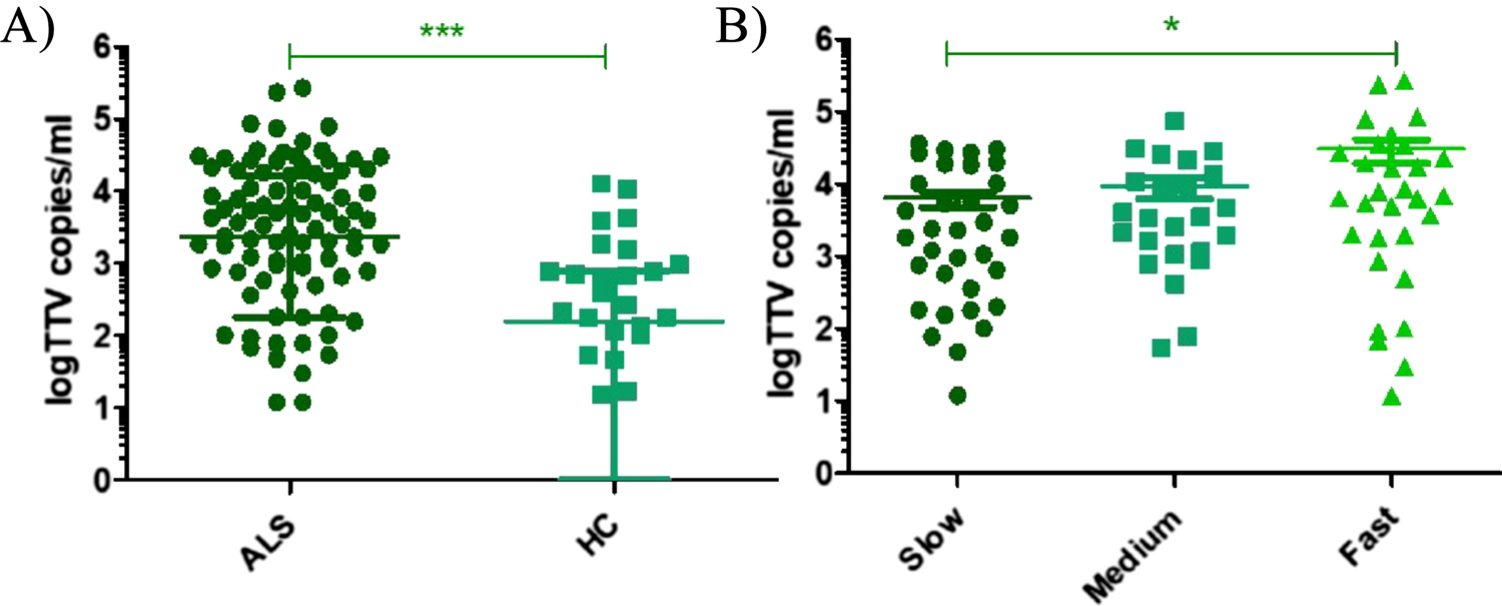
Torque teno virus abundance (copies/ml) in **A** ALS patients compared to HC; **B** ALS patients with different progression rate. ***p-value < 0.001*p-value < 0.05

The levels of TTV varied significantly among patients with different progression rate (*P* = 0.027), particularly when comparing fast progressors to slow progressors (Dunn’s multiple comparison test, *P* < 0.05) (Fig. 1). However, TTV load did not show significant differences among patients with varying disease onset types, phenotypes, or *C9ORF72* expansion (data not shown).

### Profile of the free fatty acids

Compared to healthy controls, patients with ALS exhibited a higher total FFA level (*P* < 0.0001), while their SCFA level was generally lower. Among the evaluated MCFA and LCFA, only t2-ethylhexanoic acid, octanoic acid, and octadecanoic acid showed no significant difference between ALS patients and healthy controls (Table 2). Similarly, among SCFA, only butyric acid levels did not significantly differ. The distribution of FFA across different ALS progressors groups and according to disease onset or phenotype displayed considerable variability within groups, without significant differences. Patients with a *C9ORF72* expansion showed a non-significant trend toward lower FFA levels (Supplementary Table 3).

**Table 2:** Concentrations of free fatty acids (µmol/l) determined in the serum of ALS patients and healthy controls

Analyte (µmol/l)	ALS patients (*n* = 100), median (IQR)	Healthy controls (*n* = 34), median (IQR)	*P* value (Mann–Whitney test)
FFA	951.36 (660.9–1286.7)	595.9 (415.4–736.1)	< 0.0001*
SCFA	110.8 (85.6–839.8)	263.2 (220.3–598.2)	0.019
MCFA	20.8 (14.6–26.7)	14.7 (9.4–18.7)	< 0.0001*
LCFA	711.2 (492.8–880.7)	330.0 (175.1–417.5)	< 0.0001*
Acetic acid	71.85 (71.85–336–673)	191.50 (141.71–227.71)	0.0270
Propionic acid	4.37 (4.27–6–55)	16.69 (12.5–21.45)	< 0.0001*
Butyric acid	6.60 (3.30–17.25)	5.51 (3.18–6.75)	0.014
Isobutyric acid	2.00 (1.67–3.34)	12.30 (8.42–18.18)	< 0.0001*
Isovaleric acid	1.65 (1.65–3.30)	15.78 (10.37–21.12)	< 0.0001*
2-Methylbutyric acid	1.88 (1.71–1.88)	8.92 (5.88–12.94)	< 0.0001*
Valeric acid	0.34 (0.17–0.59)	0.64 (0.34–0.85)	< 0.0001*
Hexanoic acid	0.43 (0.43–0.43)	1.90 (1.36–2.79)	< 0.0001*
Heptanoic acid	0.30 (0.15–0.45)	1.03 (0.45–3.04)	< 0.0001*
Nonanoic acid	0.33 (0.28–0.82)	0.24 (0.05–0.55)	< 0.0001*
2-Ethylhexanoic acid	3.44 (1.72–9.72)	3.56 (1.30–8.68)	0.081
Octanoic acid	1.43 (1.43–1.43)	0.77 (0.47–2.14)	0.092
Decanoic acid	1.18 (1.18–1.75)	0.41 (0.08–1.09)	< 0.0001*
Benzoic acid	8.02 (4.01–12.03)	1.65 (1.49–2.02)	< 0.0001*
Dodecanoic acid	1.29 (1.17–2.77)	2.45 (1.61–4.49)	< 0.0001*
Tetradecanoid acid	8.21 (5.20–13.12)	14.73 (8.28–24.18)	< 0.0001*
Hexadecanoic acid	541.04–399.20)	250.0 (116.25–317.22)	< 0.0001*
Octadecanoic acid	67.32 (47.36–228.49)	64.6 (38.96–91.19)	0.0310

*P*-values were calculated with Mann–Whitney test. ^*^p-value adj < 0.0022

### Multivariate analysis with the entire dataset

**Fig. 2:**
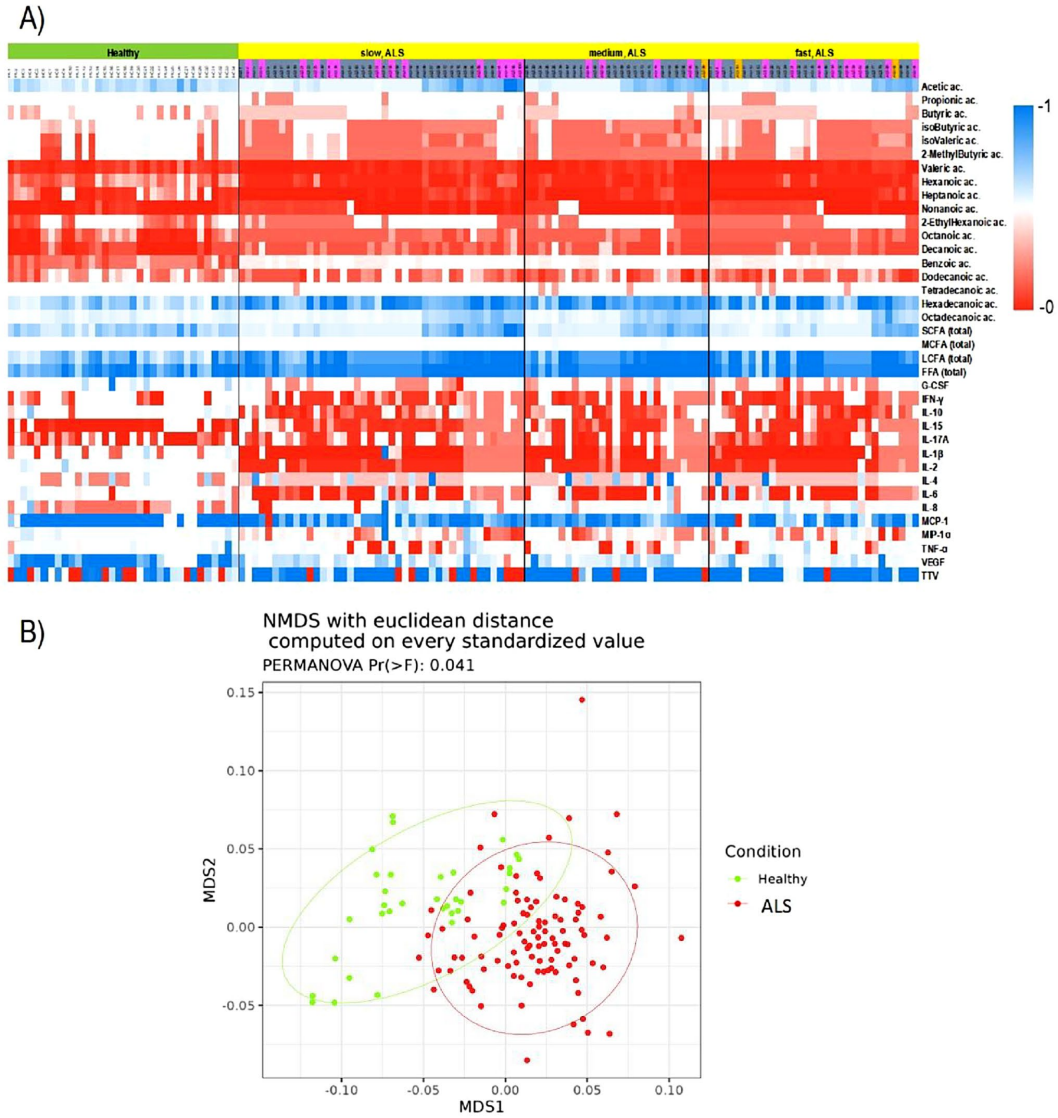
**A** Heatmap visualization based on cytokines, free fatty acids, and TTV distribution. Rows: biological variables; columns: patients. Green: HC; yellow: ALS patients; pink = bulbar onset; gray = spinal onset; orange = respiratory onset. Color key indicates metabolite expression value: blue: lowest; red: highest. **B** The sample distances among ALS patients and healthy controls have been represented through a NMDS plot with Euclidean distance on standardized values and the estimated 95% confidence interval of group centroids has been tracked as ellipses

The expression patterns of all examined biological variables are depicted in Fig. 2. To investigate the distinctions between ALS patients and healthy controls, as well as among ALS subtypes categorized by onset, phenotype, and the presence of C9ORF72 expansion, we conducted PERMANOVA tests using Euclidean distance. These tests included cytokines, fatty acids, and TTV-DNA values as independent variables. According to the PERMANOVA results, validated by the nonmetric multidimensional scaling (NMDS) analysis, there was a significant difference between ALS patients and healthy controls (Pr (> F) = 0.041) (Fig. 2B). However, it was not possible to differentiate among ALS patients based on their clinical characteristics (disease onset, phenotypes, and C9ORF72 expansion presence).

### Cluster analysis

**Fig. 3:**
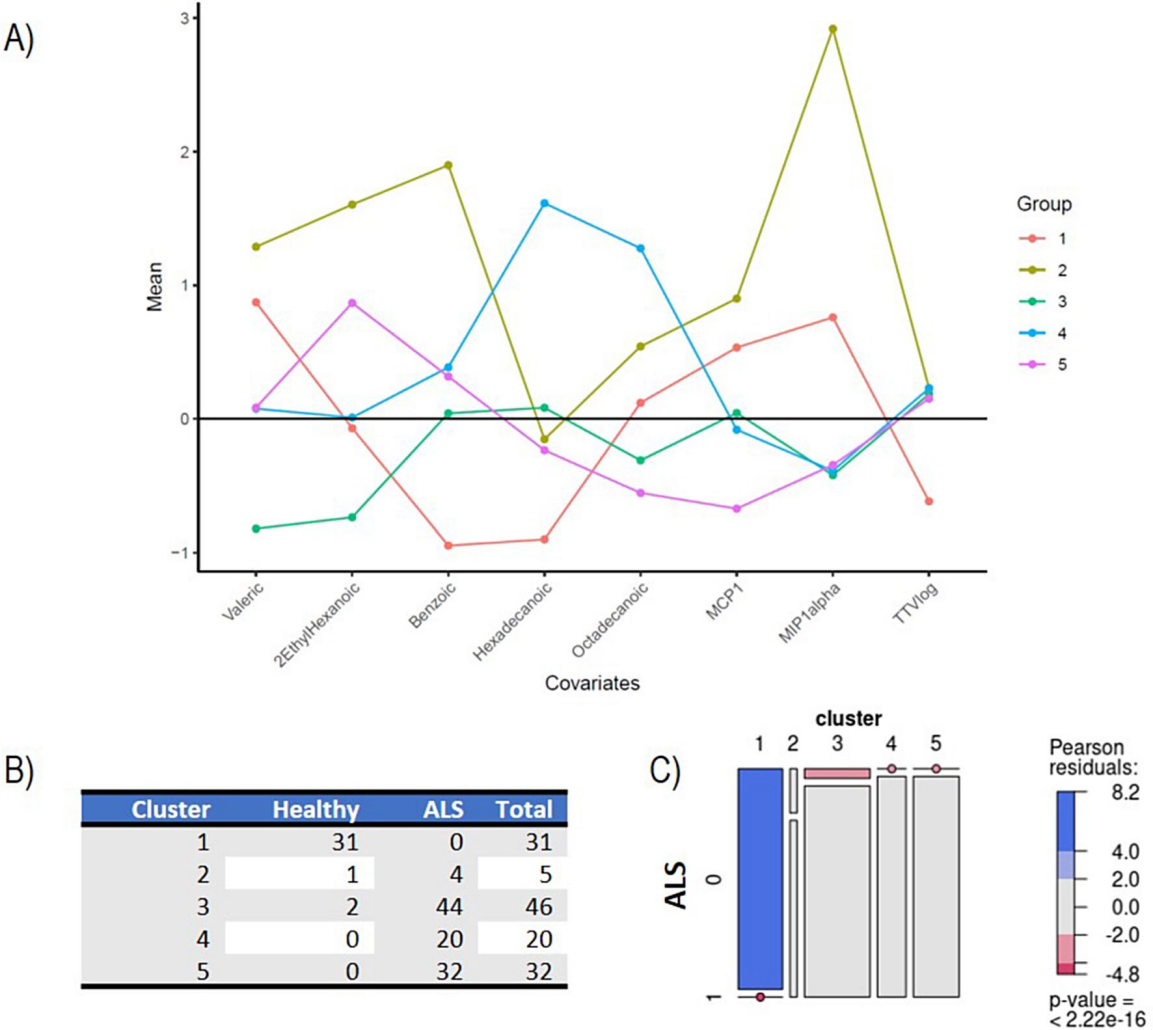
Cluster analysis **A** Mean expression levels of biological markers within the five identified clusters, after normalization to ensure zero mean and unit variance across the dataset. This normalization process allows for a straightforward comparison of biological marker expressions among clusters, using the (normalized) average expression level (represented by a horizontal black line) as a reference point. The colorful lines depict the unique “biological profile” of each cluster. **B** and **C** Contingency table and cluster membership with graphical representation of the ${\chi }^{2}$ test. Panel B shows considerable differences in the proportion of patients and healthy subjects across clusters, with a strong prevalence of healthy patients in Cluster 1. The mosaic plot represents the statistical association between cluster membership and ALS prognosis. The horizontal axis displays the distribution of cluster membership, with each segment’s width indicating the relative frequency of clusters. Vertically, within each cluster, the distribution of ALS presence is shown. The areas of rectangles represent the relative frequency of ALS patients and healthy controls in each cluster. A dot over a segment signifies no subjects with that combination of prognosis and cluster membership. Rectangles with similar heights across clusters suggest independence between prognosis and cluster membership (i.e., no association), while significantly different heights indicate an association. Colored cases indicate the strength of association, with red indicating observed frequencies smaller than expected (under the null hypothesis of independence) and blue indicating larger frequencies

To analyze the biological profile of our study participants, we utilized a Gaussian Mixture Model (GMM), selecting eight specific features based on their mean absolute deviation (MAD). These features included certain fatty acids (valeric, 2-ethylhexanoic, benzoic, hexadecanoic, and octadecanoic acids), the cytokines MCP-1 and MIP-1α, and TTV load. Our analysis identified five distinct clusters among the subjects, showing consistency in their biological features, with the number of subjects in each cluster ranging from 5 to 46 (Fig. 3A). This clustering indicates a clear partitioning of the data into distinct groups. Nonetheless, we considered Cluster 2 as a residual cluster, comprising only five subjects (one healthy control and four ALS patients without mutations), characterized by a large variance in the expression of biological variables, especially of benzoic acid and MIP-1α (Supplementary Fig. 2).

This suggests significant diversity within this cluster, indicating a lack of uniform biological patterns among its members.

Cluster 1 consists of healthy controls (91.18%), with no ALS patients, demonstrating the GMM’s efficacy in distinguishing between healthy individuals and those with ALS.

The relationship between cluster membership and ALS diagnosis was statistically significant, as shown by the ${\chi }^{2}$ test (*P* < $0.0001$) (Fig. 3B,C). While ALS patients were distributed across four clusters, no significant correlation was found between cluster membership and various clinical factors such as disease and progression rate (${\chi }^{2}$ test with *P* = $0.6866$), sex (${\chi }^{2}$ test with *P* = $0.6797$), site of onset (${\chi }^{2}$ test with *P* = $0.8483$), phenotype (${\chi }^{2}$ test with *P* = 0.6721), or the presence of *C9ORF72* expansion (${\chi }^{2}$ test with *P* = 0.0971) (Supplementary Fig. 3). This indicates that while the clusters differentiate ALS patients from healthy controls, they do not align with specific ALS clinical characteristics.

Members of Cluster 1, primarily consisting of healthy controls, consistently exhibited lower levels of TTV, benzoic acid, and hexadecanoic acid, but higher levels of MIP-1α, compared to the overall population average (Fig. 3A, Supplementary Fig. 2). When analyzing the ALS patients in Clusters 3, 4, and 5, we observed the following: (i) the levels of MIP-1α, TTV, and, to a lesser degree, benzoic acid, were similar across these clusters, all showing a markedly distinct pattern from that of Cluster 1; (ii) Cluster 3 stood out for the lower expression of valeric and 2-ethilhexanoic acids (iii) Cluster 4 exhibited higher levels of hexadecanoic and octadecanoic acids; (iv) Cluster 5 was characterized by increased levels of 2-ethylhexanoic acids and decreased levels of MCP-1 and, to a lesser extent, octadecanoic acid and MIP1-α.

To evaluate the statistical significance of our cluster-based analysis in differentiating between healthy controls and ALS patients, we conducted several statistical tests focusing on the differential expression of specific biological markers. We focused our analysis on those features for which we could formulate precise one-sided hypotheses based on the clustering results (see Fig. 3). Namely, we tested the null hypothesis of no difference between healthy controls and ALS against the alternatives of overexpression of benzoic and hexadecanoic acids and TTV in ALS and underexpression of valeric acid, MCP1, and MIP1-α in ALS.

All tests yielded highly significant results, with *p*-values < 0.0001, providing strong confidence in our findings (Supplementary Table 4).

These results are consistent with those obtained from the univariate analysis reported in Sects. 1.2–1.5, which showed significant differences in the expression of these same features between healthy controls and ALS patients.

### Survival analysis

**Fig. 4:**
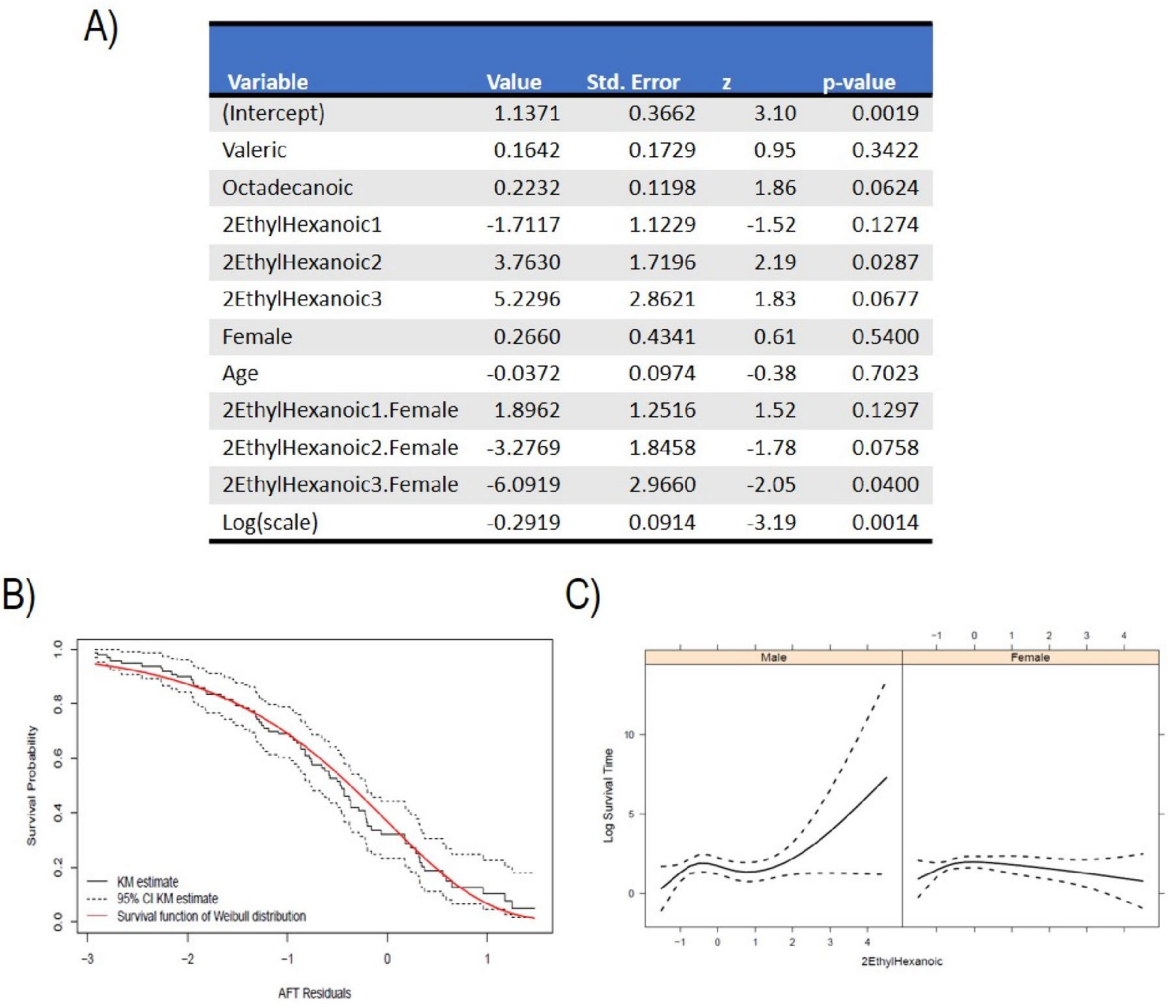
**A** Summary of the fitted survival regression model. The table reports estimated values for each predictor variable in the model, including the intercept and the log of the scale parameter. “Std. Error” is the standard deviation of the sampling distribution of the coefficient estimates, indicating the precision of the estimates. “*z*” is the *z*-statistic for each coefficient, used to test the significance of each parameter. “*p*-value” is the *p*-value associated with the *z*-value, indicating the significance of each predictor variable in the model; **B** The validity of the assumed Weibull distribution for the survival times can be assessed using residuals that account for censoring. This is done by computing the fitted model residuals and creating a Kaplan–Meier estimate. The estimated residuals and the assumed Weibull distribution are then plotted and compared to assess their fit. A good fit to the data indicates that the Weibull distribution is a suitable model for the survival times. The solid black line represents the Kaplan–Meier estimator of the residuals, with the black dotted lines representing the upper and lower 95%CI. The solid red line represents the survival probability estimated with the fitted AFT model. **C** Graphical representation of the effect of 2-ethylhexanoic acid on log survival time, considering its interaction with sex. The left panel shows the effect of 2-ethylhexanoic acid on log survival for male patients and the right panel for females. Since we modeled the effect of 2-ethylhexanoic using nonlinear terms (cubic splines), it is not constant for different expression levels. Since the biological features have been standardized, the value “0” for 2-ethylhexanoic denotes the average expression of 2-ethylhexanoic acid

Initially, we implemented a survival regression model to investigate the impact of cluster membership on tracheostomy-free survival, which was log-transformed for analysis. This model did not reveal any statistically significant association between the clustering and survival time, aligning with the observation that disease progression rates do not vary significantly across clusters (Supplementary Fig. 3). To evaluate the association between specific biological features and survival, we first utilized the sure independence screening (SIS) method (step 1), to identify the most informative features. The highest-ranking markers were valeric, 2-ethylhexanoic, and octadecanoic acids. Incorporating these acids, along with age, sex (coded as a binary variable where $1$ represents female patients), and an intercept, we ran an accelerated failure time (AFT) model assuming the error term followed a Weibull distribution. To account for potential nonlinear relationships, we allowed the impact of 2-ethylhexanoic acid on survival to be modeled nonlinearly through natural cubic splines with 3 degrees of freedom. An interaction term between 2-ethylhexanoic and sex was also included. The results are reported in Fig. 4. The AFT model demonstrated a good fit to the data (Fig. 4B) and identified significant associations: the nonlinear effects of 2-ethylhexanoic acid on survival were moderately significant (*p*-values 0.13, 0.03, 0.07 for each degree of freedom), as was the interaction between 2-ethylhexanoic acid and sex (*p*-values 0.13, 0.08, 0.04 for each degree of freedom). In an AFT model, interpreting the coefficients is typically straightforward: a one-unit increase in a covariate leads to a multiplication of the failure time by the exponent of its coefficient. For example, a one-unit increase in the level of valeric acid is associated with a 16% increase in survival time, holding other variables constant. Similarly, female patients are expected to have a 27% longer survival time compared to males, with other factors being equal.

However, the presence of interaction and nonlinear terms in our model complicates the interpretation of these coefficients. Therefore, we can graphically represent the effect of 2-ethylhexanoic acid on log-transformed survival time for both male and female patients separately, providing a visual insight into its impact.

## Discussion

In our study, we analyzed a broad spectrum of blood-based biological markers to delve into the increasingly recognized role of microbiome, metabolism, and immunity in ALS pathogenesis [47]. Our comprehensive assessment included 14 inflammatory cytokines, 18 free fatty acids (end products of human and microbial metabolism), and TTV viremia, a virome-related potential marker linked to immune system function [40].

Initial univariate analysis revealed significant differences in 30 biological markers between newly diagnosed ALS patients and controls. Specifically, we found alterations in TTV viremia, 11 of the 14 tested cytokines and 16 over 18 free fatty acids.

Most cytokines exhibited reduced levels in ALS patients compared to healthy controls, with the exception of IL-8, which was elevated. Such reductions in cytokine levels and changes in immune cell profiles are consistent with findings in ALS [5, 15, 48, 52] and similar peripheral immune dysregulations reported in other neurodegenerative diseases such as Alzheimer’s, suggesting potential commonalities in immune alteration mechanisms across different neurological disorders [35].

The observed decrease in both proinflammatory and anti-inflammatory cytokines might result from peripheral immune cell anergy or an auto-regulatory feedback aimed at mitigating neuroinflammation. Factors such as changes in blood–brain barrier (BBB) permeability, allowing the infiltration of peripheral immune cells into the central nervous system, could contribute to this immune dysregulation [35, 53]. However, the literature reports conflicting evidence, often indicating increased levels of cytokines, especially on soluble factors [60], including IL-8 [15, 25, 30, 36, 50], a chemokine involved in microglia mobilization and activation, which can exacerbate neuroinflammation and brain damage [14, 20, 49]. These discrepancies may stem from technical and methodological issues (i.e., small sample size of the analyzed cohort, selection bias, and the disease stage at which patients are studied [6]). Our previous studies, based on smaller cohorts, showed results that were not entirely consistent with the current findings [52]. In the present study, we enrolled patients shortly after diagnosis, excluding those with a history of acute infections or severe comorbidities that could independently contribute to a chronic inflammation. This careful patient selection and consideration of potential confounding factors lend additional weight to our findings, underscoring the complexity of immune dysregulation in ALS. Our study contributes to the expanding evidence on cytokine dysregulation in ALS, providing valuable insights into the intricate interplay of cytokines and immune cells in the context of ALS pathophysiology.

The observation of generally low levels of cytokines and chemokines alongside elevated TTV viremia in ALS patients, especially among those with fast and intermediate progression, points to a compromised peripheral immune response in ALS. The increased TTV load, which has been suggested as a marker of immune function, where higher levels indicate excessive immunosuppression and lower levels indicate insufficient immunosuppression in transplant patients [64], may signify an ineffective immune response to the ongoing neuroinflammation characteristic of ALS, especially in those who experience rapid disease progression. This pattern of higher TTV viremia was also found in patients with progressive multiple sclerosis compared to those with relapsing remitting forms [42], indicating a possible broader implication in neurological diseases.

Considering the noted microbiome alteration in ALS [52], a leaky gut barrier could allow the systemic spread of bacterial products and viruses. Viruses themselves may influence human health by shaping the microbiota or by direct interactions with the immune system [38], suggesting that a comprehensive analysis of the gut virome and bacteriome in ALS could provide insightful information.

Furthermore, our investigation into FFA in ALS patients and healthy controls responds to emerging evidence linking alterations in the gut microbiome to changes in plasma lipid profiles in ALS, underscoring their potential as biomarkers for the disease [29]. FFA, which serve as regulators of immunity function and metabolism, can be categorized by their carbon chain length (short, medium, long, or very-long) and saturation level. They are involved in inflammatory processes and their structural variations are linked to different biological functions, connecting lipid peroxidation and metabolism to inflammation seen in disorders such as ALS and Alzheimer’s disease [24, 62]. Our findings support previous studies showing increased levels of total and very long chain fatty acids in the blood samples of ALS patients [2, 11, 17, 27], while in addition to this, we observed elevated levels of MCFA, further highlighting the intricate interplay between lipid metabolism and disease pathogenesis in ALS. SCFA, the main microbiota-derived metabolites, known for their neuro-immunoendocrine regulatory and anti-inflammatory effects [22, 61], were found to be decreased, mirroring patterns observed in other neurological disorders [15, 32, 66].

This reinforces the potential of serum lipid levels as biomarkers and highlights the intricate relationships between metabolic changes, the microbiome, and immune responses in the context of ALS pathogenesis.

Our multivariate analysis, utilizing PERMANOVA with all the evaluated variables, successfully differentiated ALS patients from healthy controls. In addition, by applying a Gaussian Mixture Model, we identified shared biological profiles among patients. The selection of variables for this model was guided by their statistical significance, determined through the mean absolute deviation method applied on the full range of biological features. This process highlighted eight biological elements (valeric, 2-ethylhexanoic, benzoic, hexadecanoic and octadecanoic acids, MCP-1 and MIP1-α cytokines, and TTV-DNA) as differentiators between ALS patients from healthy controls. In addition, these eight biological variables organized patients into clusters with distinct characteristics. The clusters, characterized by unique metabolic signatures, did not correspond with traditional clinical ALS categories, such as onset type, phenotype, genotype, or disease progression rate. For instance, Cluster 3 exhibited low levels of valeric and 2-ethylhexanoic acids, Cluster 4 showed high levels of hexadecanoic and octadecanoic acids, and Cluster 5 had elevated 2-ethylhexanoic acid but reduced MCP-1α. This observation implies that the clusters may more accurately reflect metabolic variations in ALS rather than traditional clinical metrics [24, 34].

The distinction between these immune-metabolic clusters and classical clinical categorizations suggests the potential for identifying unique ALS biotypes, which could offer a more representation of the disease biological attributes. This perspective shift toward biological rather than clinical classification could enrich our understanding of ALS. Recognizing these groups could be instrumental in developing targeted therapeutic strategies for distinct segments within the ALS patient population. In our research, we delved into the prognostic potential of specific biological variables and uncovered a significant correlation between levels of 2-ethylhexanoic acid and survival in ALS patients, revealing a notable gender-based difference in this association. This finding emphasizes sex differences in ALS [12, 46] and introduces an additional layer of complexity in utilizing these biomarkers for disease progression prediction.

The observed “protective” effect of 2-ethylhexanoic acids in females is unprecedented and could be explained by an interaction with estrogen hormones that were found neuroprotective in ALS [63, 65]. Another possibility is represented by a sex-specific genetic architecture [10]. However, our study’s limitations must be acknowledged. By focusing solely on blood-derived biomarkers, we missed the opportunity to directly explore the gut–brain axis or to analyze gut microbiota composition differences between ALS patients and healthy controls or across the identified clusters. Consequently, we could not provide direct evidence of an imbalanced gut microbiota composition in ALS, previously established in our works [52].

Furthermore, while examining systemic immune dysregulation provides a comprehensive perspective on the immune response in ALS, an ideal approach would include analyses at the single neuronal cell level. Future studies should aim to integrate single-cell analyses to enrich our findings, offering a finer-grained insight into the cellular dynamics at play. Our study’s scope was also limited by not including patients with other neurodegenerative diseases. This is a critical aspect, as these conditions are often considered in differential ALS diagnosis. Despite our rigorous selection criteria, we could not eliminate all potential confounding factors such as diet composition [18, 52]. Nonetheless, our study successfully identified distinct clusters within the ALS patient population, characterized by unique biological profiles.

To further validate and expand our findings, future research should include a wider array of biomarkers, encompassing plasma, gut microbiome, and cellular immunity aspects, such as regulatory T cells, in larger and more diverse cohorts. In addition, our methodology was tailored specifically for TTV detection and did not account for the discovery of other potentially relevant viruses. In conclusion, our study marks a significant step forward in comprehensively analyzing serum cytokine profiles, FFA, and TTV in a substantial ALS patient cohort. We have highlighted peripheral immune deficiencies and facilitated the classification of ALS patients into distinct groups based on a combination of host (cytokines) and microbiome-related variables (TTV and SCFA).

This novel approach holds promise for better identifying ALS subgroups that may exhibit differential responses to therapeutic interventions.

### Supplementary Information

Below is the link to the electronic supplementary material.

Supplementary file1 (XLSX 58 KB)

Supplementary file2 (DOCX 399 KB)

## Data Availability

The datasets used and/or analyzed during the current study are available from the corresponding author on reasonable request.

## Abbreviations

AFT: Accelerated failure time
ALS: Amyotrophic lateral sclerosis
ALSFRS-R: ALS Functional Rating Scale-Revised
CNS: Central nervous system
ERRALS: Emilia Romagna Register of ALS
FFA: Free fatty acids
GC: Gas chromatography
GMM: Gaussian Mixture Model
MAD: Median absolute deviation
MS: Mass spectrometry
SCFA: Short chain fatty acids
TTV: Torque teno virus

## References

[1] Appel SH, Beers DR, Zhao W (2021). Amyotrophic lateral sclerosis is a systemic disease: peripheral contributions to inflammation-mediated neurodegeneration. Curr Opin Neurol.

[2] Area-Gomez E, Larrea D, Yun T, Xu Y, Hupf J, Zandkarimi F, Chan RB, Mitsumoto H (2021). Lipidomics study of plasma from patients suggest that ALS and PLS are part of a continuum of motor neuron disorders. Sci Rep.

[3] Baldi S, Menicatti M, Nannini G, Niccolai E, Russo E, Ricci F, Pallecchi M, Romano F, Pedone M, Poli G, Renzi D, Taddei A, Calabrò AS, Stingo FC, Bartolucci G, Amedei A (2021). Free fatty acids signature in human intestinal disorders: significant association between butyric acid and celiac disease. Nutrients.

[4] Banfield JD, Raftery AE (1993). Model-based Gaussian and non-Gaussian clustering. Biometrics.

[5] Beers DR, Appel SH (2019). Immune dysregulation in amyotrophic lateral sclerosis: mechanisms and emerging therapies. Lancet Neurol.

[6] Benatar M, Boylan K, Jeromin A, Rutkove SB, Berry J, Atassi N, Bruijn L (2016). ALS biomarkers for therapy development: state of the field and future directions. Muscle Nerve.

[7] Brooks BR, Miller RG, Swash M, Munsat TL (2000). El Escorial revisited: revised criteria for the diagnosis of amyotrophic lateral sclerosis. Amyotroph Lateral Scler Other Motor Neuron Disord.

[8] Cadwell K (2015). The virome in host health and disease. Immunity.

[9] Calvo A, Moglia C, Lunetta C, Marinou K, Ticozzi N, Ferrante GD, Scialo C, Sorarù G, Trojsi F, Conte A, Falzone YM, Tortelli R, Russo M, Chiò A, Sansone VA, Mora G, Silani V, Volanti P, Caponnetto C, Querin G, Monsurrò MR, Sabatelli M, Riva N, Logroscino G, Messina S, Fini N, Mandrioli J (2017). Factors predicting survival in ALS: a multicenter Italian study. J Neurol.

[10] Chapman L, Cooper-Knock J, Shaw PJ (2023). Physical activity as an exogenous risk factor for amyotrophic lateral sclerosis: a review of the evidence. Brain.

[11] Chełstowska B, Barańczyk-Kuźma A, Kuźma-Kozakiewicz M (2021). Dyslipidemia in patients with amyotrophic lateral sclerosis–a case control retrospective study. Amyotroph Lateral Scler Frontotemporal Degener.

[12] Chiò A, Logroscino G, Hardiman O, Swingler R, Mitchell D, Beghi E, Traynor BG (2009). Prognostic factors in ALS: a critical review. Amyotroph Lateral Scler.

[13] den Besten G, van Eunen K, Groen AK, Venema K, Reijngoud DJ, Bakker BM (2013). The role of short-chain fatty acids in the interplay between diet, gut microbiota, and host energy metabolism. J Lipid Res.

[14] Dheen ST, Kaur C, Ling EA (2007). Microglial activation and its implications in the brain diseases. Curr Med Chem.

[15] Ehrhart J, Smith AJ, Kuzmin-Nichols N, Zesiewicz TA, Jahan I, Shytle RD, Kim SH, Sanberg CD, Vu TH, Gooch CL, Sanberg PR, Garbuzova-Davis S (2015). Humoral factors in ALS patients during disease progression. J Neuroinflammation.

[16] Fan J, Lv J (2008). Sure independence screening for ultrahigh dimensional feature space. J R Stat Soc Ser B Stat Methodol.

[17] FernÁndez-Eulate G, Ruiz-Sanz JI, Riancho J, ZufirÍa M, GereÑu G, FernÁndez-TorrÓn R, Poza-Aldea JJ, Ondaro J, Espinal JB, GonzÁlez-ChinchÓn G, Zulaica M, Ruiz-Larrea MB, LÓpez de Munain A, Gil-Bea FJ (2020). A comprehensive serum lipidome profiling of amyotrophic lateral sclerosis. Amyotroph Lateral Scler Frontotemporal Degener.

[18] Fleischman AI, Hayton T, Bierenbaum ML (1964). Variation in composition of serum free fatty acids with dietary change under isocaloric conditions. Am J Clin Nutr.

[19] Focosi D, Macera L, Boggi U, Nelli LC, Maggi F (2015). Short-term kinetics of torque teno virus viraemia after induction immunosuppression confirm T lymphocytes as the main replication-competent cells. J Gen Virol.

[20] Franciosi S, Choi HB, Kim SU, McLarnon JG (2005). IL-8 enhancement of amyloid-beta (Abeta 1–42)-induced expression and production of pro-inflammatory cytokines and COX-2 in cultured human microglia. J Neuroimmunol.

[21] Frye BC, Bierbaum S, Falcone V, Köhler TC, Gasplmayr M, Hettich I, Dürk T, Idzko M, Zissel G, Hengel H, Müller-Quernheim J (2019). Kinetics of torque teno virus-DNA plasma load predict rejection in lung transplant recipients. Transplantation.

[22] Fung TC, Olson CA, Hsiao EY (2017). Interactions between the microbiota, immune and nervous systems in health and disease. Nat Neurosci.

[23] Gianferrari G, Martinelli I, Simonini C, Zucchi E, Fini N, Caputo M, Ghezzi A, Gessani A, Canali E, Casmiro M, De Massis P, Curro Dossi M, De Pasqua S, Liguori R, Longoni M, Medici D, Morresi S, Patuelli A, Pugliatti M, Santangelo M, Sette E, Stragliati F, Terlizzi E, Vacchiano V, Zinno L, Ferro S, Amedei A, Filippini T, Vinceti M, Mandrioli J, GROUP E (2023). Insight into elderly ALS patients in the emilia romagna region: epidemiological and clinical features of late-onset ALS in a prospective Population-Based Study. Life (Basel).

[24] Godoy-Corchuelo JM, Fernández-Beltrán LC, Ali Z, Gil-Moreno MJ, López-Carbonero JI, Guerrero-Sola A, Larrad-Sainz A, Matias-Guiu J, Matias-Guiu JA, Cunningham TJ, Corrochano S (2022). Lipid metabolic alterations in the ALS–FTD spectrum of disorders. Biomedicines.

[25] Gonzalez-Garza MT, Martinez HR, Cruz-Vega DE, Hernandez-Torre M, Moreno-Cuevas JE (2018). Adipsin, MIP-1b, and IL-8 as CSF Biomarker Panels for ALS Diagnosis. Dis Markers.

[26] Görzer I, Jaksch P, Kundi M, Seitz T, Klepetko W, Puchhammer-Stöckl E (2015). Pre-transplant plasma torque teno virus load and increase dynamics after lung transplantation. PLoS One.

[27] Goutman SA, Guo K, Savelieff MG, Patterson A, Sakowski SA, Habra H, Karnovsky A, Hur J, Feldman EL (2022). Metabolomics identifies shared lipid pathways in independent amyotrophic lateral sclerosis cohorts. Brain.

[28] Goyal NA, Berry JD, Windebank A, Staff NP, Maragakis NJ, van den Berg LH, Genge A, Miller R, Baloh RH, Kern R, Gothelf Y, Lebovits C, Cudkowicz M (2020). Addressing heterogeneity in amyotrophic lateral sclerosis CLINICAL TRIALS. Muscle Nerve.

[29] Guo K, Figueroa-Romero C, Noureldein MH, Murdock BJ, Savelieff MG, Hur J, Goutman SA, Feldman EL (2023). Gut microbiome correlates with plasma lipids in amyotrophic lateral sclerosis. Brain.

[30] Hu Y, Cao C, Qin XY, Yu Y, Yuan J, Zhao Y, Cheng Y (2017). Increased peripheral blood inflammatory cytokine levels in amyotrophic lateral sclerosis: a meta-analysis study. Sci Rep.

[31] Johnson WE, Li C, Rabinovic A (2007). Adjusting batch effects in microarray expression data using empirical Bayes methods. Biostatistics.

[32] Kaczorowska J, van der Hoek L (2020). Human anelloviruses: diverse, omnipresent and commensal members of the virome. FEMS Microbiol Rev.

[33] Kimura F, Fujimura C, Ishida S, Nakajima H, Furutama D, Uehara H, Shinoda K, Sugino M, Hanafusa T (2006). Progression rate of ALSFRS-R at time of diagnosis predicts survival time in ALS. Neurology.

[34] Kirk SE, Tracey TJ, Steyn FJ, Ngo ST (2019). Biomarkers of metabolism in amyotrophic lateral sclerosis. Front Neurol.

[35] Koca S, Kiris I, Sahin S, Cinar N, Karsidag S, Hanagasi HA, Yildiz GB, Tarik Baykal A (2022). Decreased levels of cytokines implicate altered immune response in plasma of moderate-stage Alzheimer's disease patients. Neurosci Lett.

[36] Kuhle J, Lindberg RLP, Regeniter A, Mehling M, Steck AJ, Kappos L, Czaplinski A (2009). Increased levels of inflammatory chemokines in amyotrophic lateral sclerosis. Eur J Neurol.

[37] Leek JT JW, Parker HS, Fertig EJ, Jaffe AE, Zhang Y, Storey JD, Torres LC (2021) sva: surrogate variable analysis. R package version 3.40.0

[38] Li Y, Fu X, Ma J, Zhang J, Hu Y, Dong W, Wan Z, Li Q, Kuang Y-Q, Lan K, Jin X, Wang J-H, Zhang C (2019). Altered respiratory virome and serum cytokine profile associated with recurrent respiratory tract infections in children. Nat Commun.

[39] Ma Q, Xing C, Long W, Wang HY, Liu Q, Wang RF (2019). Impact of microbiota on central nervous system and neurological diseases: the gut-brain axis. J Neuroinflammation.

[40] Maggi F, Bendinelli M, de Villiers E-M, Hausen HZ (2009). Immunobiology of the torque teno viruses and other Anelloviruses. TT viruses the still elusive human pathogens.

[41] Maggi F, Focosi D, Statzu M, Bianco G, Costa C, Macera L, Spezia PG, Medici C, Albert E, Navarro D, Scagnolari C, Pistello M, Cavallo R, Antonelli G (2018). Early post-transplant torquetenovirus viremia predicts cytomegalovirus reactivations in solid organ transplant recipients. Sci Rep.

[42] Mancuso R, Saresella M, Hernis A, Agostini S, Piancone F, Caputo D, Maggi F, Clerici M (2013). Torque teno virus (TTV) in multiple sclerosis patients with different patterns of disease. J Med Virol.

[43] Mandrioli J, Biguzzi S, Guidi C, Sette E, Terlizzi E, Ravasio A, Casmiro M, Salvi F, Liguori R, Rizzi R, Pietrini V, Borghi A, Rinaldi R, Fini N, Chierici E, Santangelo M, Granieri E, Mussuto V, De Pasqua S, Georgoulopoulou E, Fasano A, Ferro S, D'Alessandro R (2015). Heterogeneity in ALSFRS-R decline and survival: a population-based study in Italy. Neurol Sci.

[44] Mandrioli J, Malerba SA, Beghi E, Fini N, Fasano A, Zucchi E, De Pasqua S, Guidi C, Terlizzi E, Sette E, Ravasio A, Casmiro M, Salvi F, Liguori R, Zinno L, Handouk Y, Rizzi R, Borghi A, Rinaldi R, Medici D, Santangelo M, Granieri E, Mussuto V, Aiello M, Ferro S, Vinceti M (2018). Riluzole and other prognostic factors in ALS: a population-based registry study in Italy. J Neurol.

[45] Mandrioli J, Zucchi E, Martinelli I, Van der Most L, Gianferrari G, Moglia C, Manera U, Solero L, Vasta R, Canosa A, Grassano M, Brunetti M, Mazzini L, De Marchi F, Simonini C, Fini N, Tupler R, Vinceti M, Chiò A, Calvo A (2023). Factors predicting disease progression in C9ORF72 ALS patients. J Neurol.

[46] Manjaly ZR, Scott KM, Abhinav K, Wijesekera L, Ganesalingam J, Goldstein LH, Janssen A, Dougherty A, Willey E, Stanton BR, Turner MR, Ampong M-A, Sakel M, Orrell RW, Howard R, Shaw CE, Leigh PN, Al-Chalabi A (2010). The sex ratio in amyotrophic lateral sclerosis: a population based study. Amyotroph Lateral Scler.

[47] Mazzini L, De Marchi F, Niccolai E, Mandrioli J, Amedei A, Araki T (2021). Gastrointestinal status and microbiota shaping in amyotrophic lateral sclerosis: a new frontier for targeting?. Amyotrophic lateral sclerosis.

[48] McCombe PA, Lee JD, Woodruff TM, Henderson RD (2020). The peripheral immune system and amyotrophic lateral sclerosis. Front Neurol.

[49] McLarnon JG (2012). Microglial chemotactic signaling factors in Alzheimer's disease. Am J Neurodegener Dis.

[50] Mennini T, Giordano L, Mengozzi M, Ghezzi P, Tonelli R, Mantegazza R, Silani V, Corbo M, Lunetta C, Beghi E (2009). Increased Il-8 levels in the cerebrospinal fluid of patients with amyotrophic lateral sclerosis. Eur J Inflamm.

[51] Neil JA, Cadwell K (2018). The intestinal virome and immunity. J Immunol.

[52] Niccolai E, Di Pilato V, Nannini G, Baldi S, Russo E, Zucchi E, Martinelli I, Menicatti M, Bartolucci G, Mandrioli J, Amedei A (2021). The gut microbiota-immunity axis in als: a role in deciphering disease heterogeneity?. Biomedicines.

[53] Ott BR, Jones RN, Daiello LA, de la Monte SM, Stopa EG, Johanson CE, Denby C, Grammas P (2018). Blood-cerebrospinal fluid barrier gradients in mild cognitive impairment and alzheimer's disease: relationship to inflammatory cytokines and chemokines. Front Aging Neurosci.

[54] Park J, Kim M, Kang SG, Jannasch AH, Cooper B, Patterson J, Kim CH (2015). Short-chain fatty acids induce both effector and regulatory T cells by suppression of histone deacetylases and regulation of the mTOR-S6K pathway. Mucosal Immunol.

[55] Rezahosseini O, Drabe CH, Sørensen SS, Rasmussen A, Perch M, Ostrowski SR, Nielsen SD (2019). Torque-Teno virus viral load as a potential endogenous marker of immune function in solid organ transplantation. Transplant Rev.

[56] Rooks MG, Garrett WS (2016). Gut microbiota, metabolites and host immunity. Nat Rev Immunol.

[57] Ruiz P, Martínez-Picola M, Santana M, Muñoz J, Pérez-del-Pulgar S, Koutsoudakis G, Sastre L, Colmenero J, Crespo G, Navasa M (2019). Torque teno virus is associated with the state of immune suppression early after liver transplantation. Liver Transpl.

[58] Schito P, Ceccardi G, Calvo A, Falzone YM, Moglia C, Lunetta C, Marinou K, Ticozzi N, Scialo C, Sorarù G, Trojsi F, Conte A, Tortelli R, Russo M, Zucchi E, Pozzi L, Domi T, Carrera P, Agosta F, Quattrini A, Fazio R, Chiò A, Sansone VA, Mora G, Silani V, Volanti P, Caponnetto C, Querin G, Tedeschi G, Sabatelli M, Logroscino G, Messina S, Mandrioli J, Riva N, Filippi M (2020). Clinical features and outcomes of the flail arm and flail leg and pure lower motor neuron MND variants: a multicentre Italian study. J Neurol Neurosurg Psychiatry.

[59] Scrucca L, Fop M, Murphy TB, Raftery AE (2016). mclust 5: clustering, classification and density estimation using gaussian finite mixture models. R J.

[60] Staats KA, Borchelt DR, Tansey MG, Wymer J (2022). Blood-based biomarkers of inflammation in amyotrophic lateral sclerosis. Mol Neurodegener.

[61] Stilling RM, van de Wouw M, Clarke G, Stanton C, Dinan TG, Cryan JF (2016). The neuropharmacology of butyrate: the bread and butter of the microbiota-gut-brain axis?. Neurochem Int.

[62] Tracey TJ, Kirk SE, Steyn FJ, Ngo ST (2021). The role of lipids in the central nervous system and their pathological implications in amyotrophic lateral sclerosis. Semin Cell Dev Biol.

[63] Trojsi F, D’Alvano G, Bonavita S, Tedeschi G (2020). Genetics and sex in the pathogenesis of amyotrophic lateral sclerosis (ALS): is there a link?. Int J Mol Sci.

[64] van Rijn A, Roos R, Dekker F, Rotmans J, Feltkamp M (2023). Torque teno virus load as marker of rejection and infection in solid organ transplantation–A systematic review and meta-analysis. Rev Med Virol.

[65] Vegeto E, Villa A, Della Torre S, Crippa V, Rusmini P, Cristofani R, Galbiati M, Maggi A, Poletti A (2020). The role of sex and sex hormones in neurodegenerative diseases. Endocr Rev.

[66] Virgin HW, Wherry EJ, Ahmed R (2009). Redefining chronic viral infection. Cell.

[67] Wei LJ (1992). The accelerated failure time model: a useful alternative to the Cox regression model in survival analysis. Stat Med.

[68] Zhang H, Chen Y, Wang Z, Xie G, Liu M, Yuan B, Chai H, Wang W, Cheng P (2022). Implications of gut microbiota in neurodegenerative diseases. Front Immunol.

