# Supplementary material for: Amyotrophic lateral sclerosis stratification: unveiling patterns with virome, inflammation, and metabolism molecules
Source: J Neurol. 2024 Apr 21;271(7):4310–25. doi: 10.1007/s00415-024-12348-7 (PMC11233352; doi:10.1007/s00415-024-12348-7)
Supplement: Supplementary file 2 — Supplementary file2 (DOCX 399 KB) [file 415_2024_12348_MOESM2_ESM.docx]

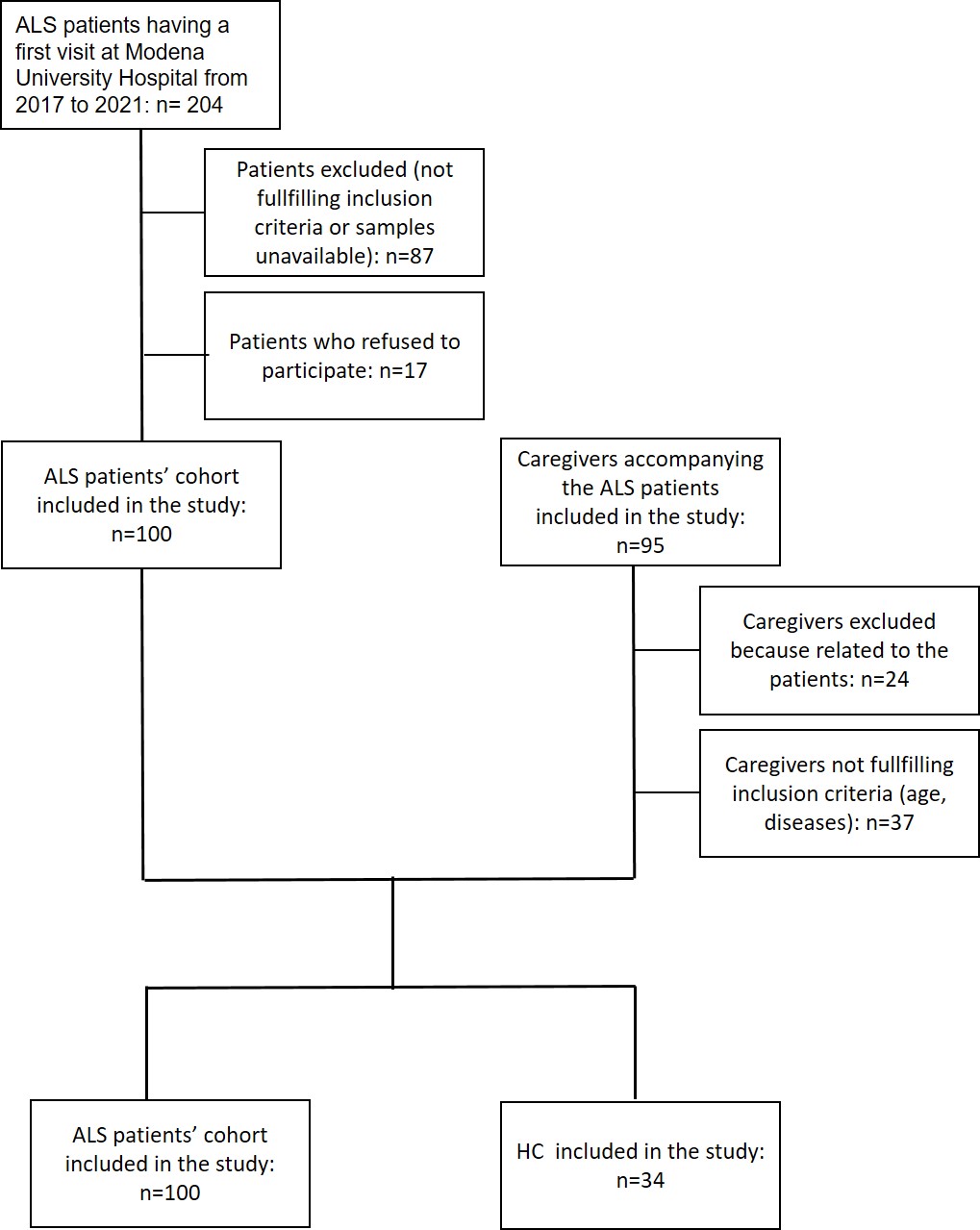


**Supplementary Figure 1**. ALS Patient Enrollment: Following STROBE Guidelines for Transparent Reporting of Observational Studies in Epidemiology


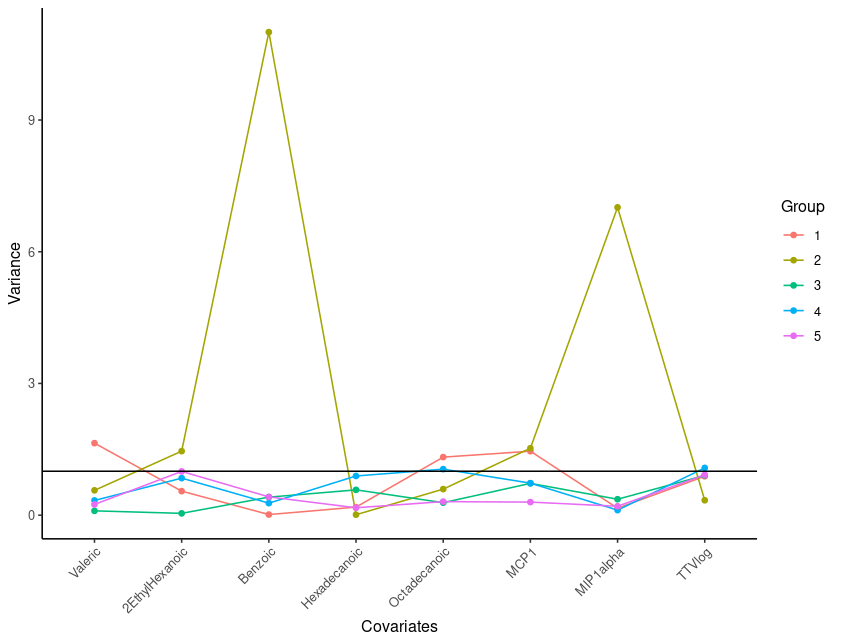


**Supplementary Figure 2.** Within cluster variance of biological features. The figure shows the variance of biological features across each identified cluster from the analysis. Before analysis, the biological features were standardized to have zero mean and unitary variance. Cluster 2 manifests large within-cluster variance, particularly concerning the expression of Benzoic Acid and MIP-1α. This observation implies that the patients included in Cluster 2 exhibit significant heterogeneity and lack coherent patterns. Cluster 2 is therefore deemed as residual.

**
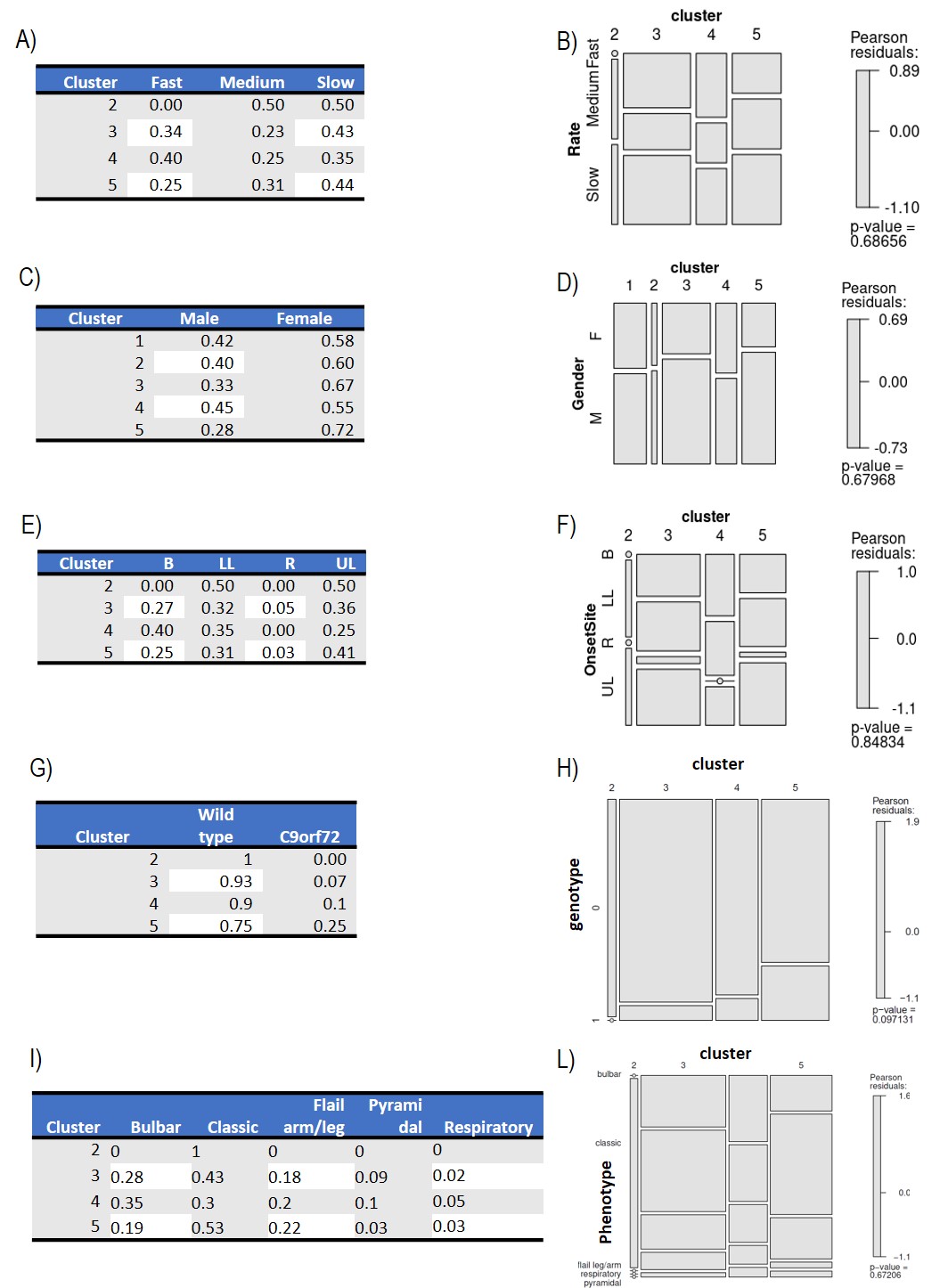
**

**Supplementary figure 3.**

Contingency table and cluster membership with graphical representation of the $\chi^{2}$ test. The mosaic plots help to visualize the statistical association between two categorical variables, in our case, cluster membership and progression rate (A, B), sex (C, D), site of onset (E, F), phenotypes (G, H) and genotype (I, L). Cluster 1 is not represented because it does not contain ALS patients. The frequency distribution of the first variable (cluster membership) is represented on the horizontal axis. The relative frequency of each cluster is proportional to the width of the corresponding segment on the x-axis. The joint frequency distribution (the relative frequencies of each combination of the two categorical variables) is represented by the areas of the corresponding rectangles. The dot over the segment in place of a rectangle means that no subject shows that combination of factors. Within each level of the first variable, the frequency distribution of the second variable is shown vertically. If these conditional distributions look similar, there is no association between the two factors. By contrast, significantly different conditional distributions indicate an association. Panels B, D, F, H, L: do not display a significant difference in the proportion of progression rate, sex, site of onset, genotype, and phenotype across clusters. Results of $\chi^{2}$ test to analyse the association between cluster membership and features are also displayed.
